# Editorial: Impact of the inflammatory microenvironment on immune infiltration in colorectal and liver cancers: insights for new immunotherapeutic strategies

**DOI:** 10.3389/fonc.2025.1691488

**Published:** 2025-10-27

**Authors:** Yuchuan Jiang, Kai Xiong

**Affiliations:** Department of Gastroenterology, The Second Affiliated Hospital, Jiangxi Medical College, Nanchang University, Jiangxi, China

**Keywords:** hepatocellular cancer, inflammatory microenvironment, colorectal cancer, immunotherapeutic strategies, immune infiltration

Together, colorectal cancer and liver cancer account for a substantial and increasing global cancer burden (1, 2). Over the past decade, immunotherapy has made significant progress in treating multiple malignancies, including these two diseases (3). However, the clinical benefit depends heavily on the immunological phenotype of the tumor microenvironment (TME). Commonly used categories include immune-enriched (activation), fibrotic/immune-enriched (immune residence), fibrotic (immune suppression), and immune-depleted (immune exclusion) states (4). To overcome primary and acquired resistance, combination regimens that pair immune checkpoint inhibitors (ICIs) with TME-remodeling agents have become a central strategy (5). Inflammation drives the initiation and evolution of colorectal and liver cancers. Beyond mutagenesis, chronic low-grade inflammation nonspecifically activates T cells, promotes exhaustion and anergy, recruits and skews immunosuppressive cells, and ultimately establishes tumor immune tolerance (6, 7). Clarifying how inflammatory cues enforce tolerance and building prognostic models anchored in inflammation-linked immune indices may improve outcomes and quality of life for patients with colorectal and liver cancers.

This Research Topic brings together original studies and a systematic review that advance mechanistic insight and translational readiness across spatial pathology, epigenetics, niche mapping, and functional prognostics. Chen et al. introduced tumor infiltration proportion within lymph nodes (TIPLN) as a pragmatic, pathology-embedded spatial biomarker for N1 colorectal cancer. Across training and validation cohorts, per−patient maximal TIPLN independently predicted overall survival, and, when incorporated into a nomogram, TIPLN improved discrimination, reclassification, and net clinical benefit—operationalizing regional niche remodeling for postoperative risk stratification and prospective trial enrichment. Liang et al. delineated an epigenetic–immune escape axis in hepatocellular carcinoma centered on GINS1. Using transcriptomic, proteomic, and immunohistochemical evidence, the authors demonstrated GINS1 upregulation, site-specific methylation associations with survival, broad coupling to m6A regulators, and positive correlations with multiple inhibitory checkpoints, nominating GINS1 and its methylome as companion biomarkers and supporting epigenetic–checkpoint combinations in HCC. He et al. provided a decade-spanning bibliometric synthesis of colorectal cancer TME research, documenting the field’s rapid growth since 2019 and convergent hotspots in ICI therapeutics, CAF heterogeneity and macrophage polarization, intestinal microbiota, colorectal liver metastasis, drug resistance, and single-cell/spatial multi-omics. Their analysis identified China and the United States as major collaboration hubs and underscores the need for higher research quality, standardization, and deeper international partnerships. Ma et al. integrated 14 programmed cell death (PCD) pathways to derive a three−gene signature—FABP4 as a risk gene and AQP8 and NAT1 as protective—that robustly stratifies prognosis across multiple cohorts and aligns with an “immune−inflamed” versus “stroma−dominant” niche dichotomy. Low−risk tumors showed greater infiltration by B cells, CD4+ and CD8+ T cells, NK cells, and M1 macrophages; reduced stromal and endothelial signals; higher predicted and observed ICI responsiveness; and greater sensitivity to first−line chemotherapy. Functional assays further showed that NAT1 overexpression augments apoptosis, reinforcing the biological plausibility of the authors’ findings.

Together, these studies outline a concise framework linking inflammation to niche states and clinical outcomes. Companion diagnostics should integrate spatial pathology (TIPLN, TLS maturation, immune topology, and CAF/stroma/vascular scores), molecular markers (GINS1 expression and methylation and multi−PCD scores), and dynamic readouts (ctDNA, circulating immune profiling, and radiomics fused with spatial omics). Therapeutically, niche−decompressing combinations—ICIs with anti−TGF−β or CSF1R blockade— should be prioritized with selective addition of LAG−3/TIGIT inhibition; epigenetic–ICI regimens should be added for GINS1/methylation−defined subsets; metabolic and vascular normalization should be paired with ICIs; and microbiome interventions, particularly for colorectal liver metastasis, should be applied. The perioperative and neoadjuvant windows are opportune times to reprogram niches and establish durable immune memory. The next steps include standardizing TIPLN and PCD cutoffs through multicenter, real−world validation; deploying integrated, single−cell/spatial, multi−omics with harmonized digital pathology to reduce bias; testing causality in organoid–immune co−cultures and humanized models; and designing trials that are stratified by spatial and molecular niche fingerprints, with biomarker−guided monitoring to balance efficacy and toxicity—especially in cirrhosis and microbiome−modulating contexts.
